# Measurement of the Neutron Lifetime Using a Gravitational Trap and a Low-Temperature Fomblin Coating

**DOI:** 10.6028/jres.110.049

**Published:** 2005-08-01

**Authors:** A. Serebrov, V. Varlamov, A. Kharitonov, A. Fomin, Yu. Pokotilovski, P. Geltenbort, J. Butterworth, I. Krasnoschekova, M. Lasakov, R. Tal’daev, A. Vassiljev, O. Zherebtsov

**Affiliations:** Petersburg Nuclear Physics Institute, Russian Academy of Sciences, 188300 Gatchina, Leningrad District, Russia; Joint Institute for Nuclear Research, Dubna, Moscow Region, 141980, Russia; Institut Max von Laue—Paul Langevin, B.P. 156, 38042 Grenoble Cedex 9, France; Petersburg Nuclear Physics Institute, Russian Academy of Sciences, 188300 Gatchina, Leningrad District, Russia

**Keywords:** neutron lifetime, ultracold neutrons

## Abstract

We present a new value for the neutron lifetime of 878.5 ± 0.7_stat._ ± 0.3_syst_. This result differs from the world average value by 6.5 standard deviations and by 5.6 standard deviations from the previous most precise result. However, this new value for the neutron lifetime together with a *β*-asymmetry in neutron decay, *A*_0_, of −0.1189(7) is in a good agreement with the Standard Model.

## 1. Experimental Set-Up

We present a new value for the neutron lifetime of 878.5 ± 0.7_stat._ ± 0.3_syst_. This result differs from the world average value by 6.5 standard deviations and by 5.6 standard deviations from the previous most precise result [1]. However, this new value for the neutron lifetime together with a *β*-asymmetry in neutron decay, *A*_0_, of −0.1189(7) [2] is in a good agreement with the Standard Model.

**Fig. 1:**
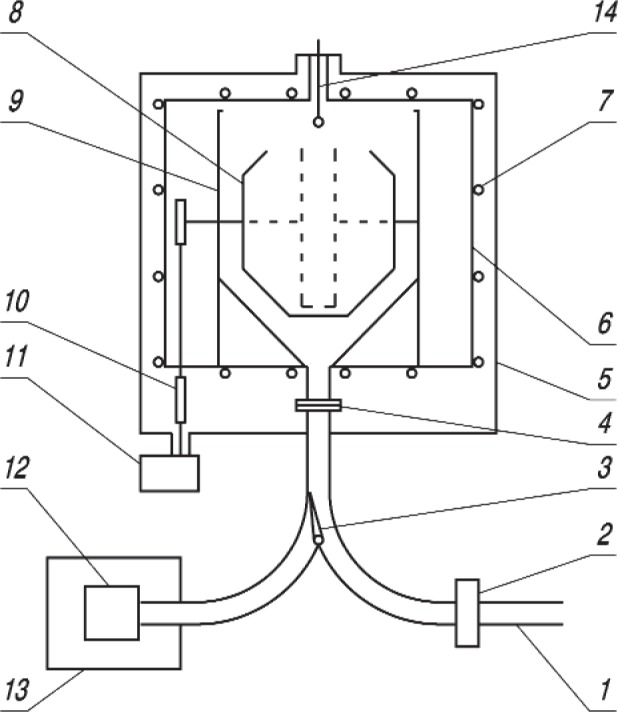
The Scheme of “Gravitrap”, the gravitational UCN storage system. 1: neutron guide from UCN Turbine; 2: UCN inlet valve; 3: beam distribution flap valve (shown in the filling position); 4: connection unit; 5: “high” vacuum volume; 6: “rough” vacuum volume; 7: cooling coils; 8: UCN storage trap (the narrow cylindrical trap is shown by a dashed line); 9: cryostat; 10: mechanics for trap rotation; 11: stepping motor; 12: UCN detector; 13: detector shielding; 14: evaporator.

The present measurements were carried out at the high flux reactor at ILL in Grenoble, France using the PF2/MAM instrument; the experimental set-up is sketched in Fig. 1. It is a gravitational trap for UCN and at the same time it can be used as a differential gravitational spectrometer. Therefore the distinguishing feature of this experiment is the ability to measure the UCN energy spectrum after its storage in the trap.

The UCN storage trap 8 is mounted inside a cryostat vacuum vessel 9. The trap 8 has a window that can be rotated about a horizontal axis so that UCN are held in the trap by gravity when the trap window is in its upper position.

UCNs enter the trap via the neutron guide 1, the opened UCN inlet valve 2 and the distribution flap valve 3. Filling takes place when the trap window is in the down position. After the trap is filled it is rotated into the up position.

A double walled vacuum system was used with separate “high” 6 and “rough” 5 vacuum vessels. The pressure in the cryostat vacuum vessel was 5 × 10^−6^ mbar; at this pressure, the residual gas has a small effect (0.4 s) on storage time for the UCN in the trap. To cool the trap we used heat exchange between the trap and the cryostat tank; to do this helium gas was flowed through the cryostat vacuum vessel and removed before carrying out the neutron lifetime measurements.

The height of the trap window relative to the trap bottom defines the maximum energy of UCN that can be held in the trap. Different window heights correspond to different cut-off energies for the UCN spectrum. Such a rotateable trap is a gravitational spectrometer. The spectral dependence of the storage time can be measured by turning the trap window downward in steps. The trap was kept in each intermediate position during 100 s to 150 s to detect most of the UCN in the corresponding energy range. The same procedure also measures the spectrum of the trapped UCN.

The neutron lifetime is measured with the size extrapolation method using two sizes of UCN trap. The first is a quasi-spherical trap consisting of a cylinder about 84 cm in diameter and 26 cm wide, capped by two truncated cones each 22 cm high, with small diameters of 42 cm and the second a 76 cm diameter cylindrical trap that was 14 cm long between its end faces. The second trap increases the neutron collision rate with the walls of the trap by a factor of about 2.5. The narrow cylindrical trap is shown in Fig. 1 by a dashed line.

**Fig. 2:**
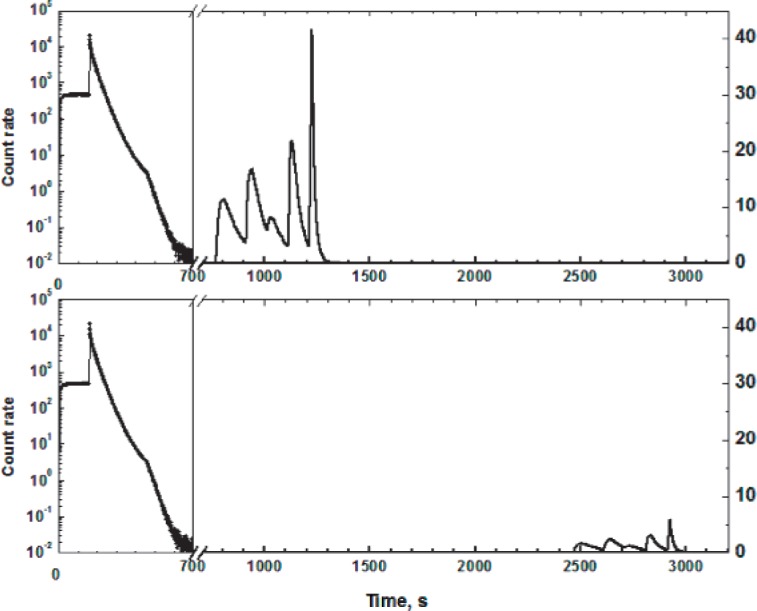
Time diagrams of the storage cycle for two different holding times.

A typical count rate diagram during the UCN storage cycle is shown in Fig. 2. First the trap is filled with UCN with the hole in the down position. Then the trap is rotated to the monitoring position where the height of the trap window is 10 cm lower than when in the holding position with the hole upward. The filling process can be observed by means of the detector *12* through the slits in the distribution valve. When the trap reaches the monitor height the distributive valve is changed to the detection position. The trap is kept in the monitoring position for 300 s. During this period the neutrons whose energy exceeds the gravitational barrier of the trap escape. Then the trap is rotated to the holding position. Overall this process takes about 500 s and the counting rate is shown on a logarithmic scale on the left side of Fig. 2; the counting rate for the subsequent procedures (700 s to 3160 s) is shown on a linear scale on the right side, in order to show more details. After a short (top part of Fig. 2) or long (bottom part of Fig. 2) holding time, the trap is rotated to five successive positions and is held in each position for 100 s to 150 s in order to count UCN. The neutrons that are counted after each rotation have a different average energy. When the UCN trap is empty the background measurement is started. The angle positions of the trap are: *θ* = 30°, (monitoring position), *θ* = 40°, *E*_UCN_ = 58 cm;*θ* = 50°, *E*_UCN_ = 52 cm; *θ* = 60°, *E*_UCN_ = 46 cm; *θ* = 75°, *E*_UCN_ = 39 cm; *θ* = 180°, *E*_UCN_ = 25 cm. The angles were chosen so as to obtain similar counts for each portion of the UCN spectrum (unfortunately, the third portion of was not successfully optimised as may be seen in Fig. 2).

## 2. The Low-Temperature Fomblin Coating of Traps

In the experiment, we have used a new type of wall coating, a low-temperature Fomblin (LTF) that can be evaporated onto the surface in vacuum. This perfluorinated oil has a composition containing only C, O, and F and thus a low neutron capture cross section. Earlier investigations [3] of several types of LTF found that quasi-elastic UCN scattering and thermal inelastic scattering are significantly lower at temperatures below −120 °C than for ordinary Fomblin oil close to room temperature. The quasi-elastic UCN scattering is suppressed completely below −120 °C [3] and the expected UCN loss coefficient *η* due to up-scattering is about 2 × 10^−6^ [4, 5].

To test the oil film quality we measured the UCN lifetime in a titanium-coated Cu trap with various coating of LTF. Titanium has a negative scattering length and UCN cannot be held in this trap when uncoated. The trap was a cylinder of diameter of 76 cm and length 50 cm. A stable storage time *τ_st._* = 867 ± 2 s was reached after several LTF evaporations (total thickness of 15 µm) at a wall temperature of between −140 °C and −150 °C, then warming the trap up to room temperature and finally re-cooling to −160 °C. Due to this process, the oil at room temperature filled all the gaps and cracks in the wall and formed a perfect surface. Additionally, the LTF has been degassed in a thin layer at room temperature. The coating is very stable and no significant change of storage time was found over an 8 days observation period. Further evaporation did not change the storage time.

For the final measurements we used Be-coated traps (quasi-spherical and narrow cylindrical). Since Be is a good UCN reflector, we can use even lower wall temperatures, making the development of any micro-cracks in the coating less detrimental to the storage time. Using the Be-coated trap we studied the temperature dependence of the storage time for the quasi-spherical trap with an LTF coating. The LTF was deposited at −140 °C, and then the trap was slowly warmed up to −50 °C and finally cooled again to −160 °C. In this way we covered layer defects by the oil when it was sufficiently liquid. Following this temperature cycle we obtained a higher storage time, 872 ± 1.5 s, than immediately after evaporation (850 ± 1.8 s). Taking into account the different sizes of the Ti- and Be-traps we find no difference in the reflection loss coefficient for the two kinds of sub-layer. This indicates that reproducible oil films were obtained independently of the substrate material.

The stability and integrity of the coating of the different traps is the most important condition for validity of the size extrapolation method for the neutron lifetime measurement. Therefore the quality of the LTF coatings was verified many times during the course of the measurements. The measurements were carried out after new evaporations, warming and cooling, new evaporations and so on. As a final improvement, the pressure in the trap was reduced from 5 × 10^−6^ mbar to 3 × 10^−7^ mbar by installing a LHe cryopump near the storage volume. The storage times over the course of the neutron lifetime experiment agreed within about 1 s for the wide trap and by a little bit more for the narrow trap. Therefore we have no reason to consider different loss factors, *η*, for the different traps.

## 3. Results of Measurements and Extrapolation to the Neutron Lifetime

**Fig. 3:**
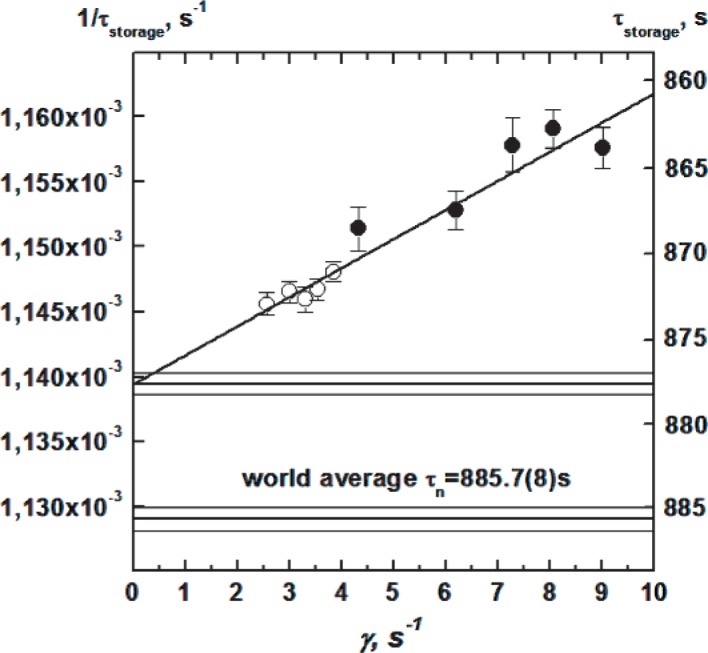
Result of extrapolation to the neutron lifetime using joint energy and the size extrapolation method. Measurements made with a spherical (open circles) and cylindrical (filled circles) traps.

The results of measurements of the UCN storage time for different energy intervals and for different traps (wide and narrow) are presented in Fig. 3 as a function of effective frequency of collisions *γ*. The extrapolation of all data to the neutron lifetime gives a value of (877.60 ± 0.65) s with a *χ*^2^ of 0.95. This means that joint extrapolation is possible. Nevertheless, we have done the energy extrapolation for each trap and on combining both results we obtain (875.55 ± 1.6) s.

For the size extrapolation method we have to connect the values for the different traps for the same UCN energy interval, and then to calculate the average value of all determinations of the neutron lifetime. The average value of the neutron lifetime from the size extrapolation method is (878.07 ± 0.73) s.

The results obtained from the two methods are different by 1.5 standard deviations. The loss factor, *η* = 2 × 10^−6^, obtained in this experiment is in agreement with that obtained in a transmission experiment [5]. As the final value for the neutron lifetime we prefer to use the result from the size extrapolation as this has a rather weak dependence on the energy dependence of losses *µ* (*E*) and we consider it to be the more reliable.

## 4. Monte Carlo Simulation of the Experiment and Systematic Errors

To both estimate the accuracy of and to check the reliability of the size extrapolation method using the calculated *γ*-function, we have used a Monte Carlo (MC) simulation of the experiment.

In the MC-simulations the behaviour of neutrons has been described taking into account the gravitational field, the form of the storage traps, the geometry of the secondary vessel and the UCN guide for transporting the UCN to the detector. As a result we can simulate directly the measurements and obtain the time diagram as shown in Fig. 2. The UCN storage times in the traps have been calculated in the same way as done in the experiment, and the extrapolation to the neutron lifetime using the calculated *γ*-function made as well. The single free parameter in the MC-simulation is the coefficient of diffuse scattering at the interaction with the trap surface. A comparison between the results of MC simulations with various values for the diffuse scattering probability and the experimental results in Fig. 2, allows us to conclude that the probability of diffuse scattering of UCN on the LTF coating is 10 % or more.

The final simulation of the experiment was done using probabilities of diffuse reflection of 10 % and 1 %. The simulated storage times were extrapolated to the neutron lifetime for both the wide cylindrical and the narrow cylindrical traps and for the five different UCN energy intervals of the experiment. To simplify the MC calculations we used a cylinder rather than the quasi-spherical form for the wide trap. The final analysis of the simulated measurements reproduced the neutron lifetime value assumed in the calculation with an uncertainty of ± 0.236 s. This uncertainty is limited by the uncertainty of the MC calculation. That is, the systematic uncertainty of the size extrapolation method using the calculated *γ*-function is ± 0.236 s.

## 5. The Influence of the Residual Gas for UCN Storage

On the level of uncertainty for neutron lifetime measurements of about 1 s, the influence of residual gas at a pressure of 5 × 10^−6^ mbar is already important. This correction cannot be measured directly, for instance by improving the vacuum by one order of magnitude, because the expected effect is less than the uncertainty. Instead, we increased the residual gas pressure to 8 × 10^−4^ mbar, making the (*pτ*)–parameter for residual gas 9.5 mbar × s and obtained a calculated correction to the storage time of (0.4 ± 0.02) s. It does not depend on UCN energy, so this correction can be used for the neutron lifetime result.

## 6. Final Result for the Neutron Lifetime and the List of Systematic Corrections and Uncertainties

Values of systematic effects with their uncertainties are shown in Table 1. The main contribution to uncertainty we have is due to measurement statistics. The next largest is the uncertainty in the calculation of the function *γ*. The contributions due to influence of the shape of the *µ*(*E*)-function and uncertainty of UCN spectrum are considerably less; these were estimated by means of variation of their parameters within the uncertainty allowed by the experimental data. Thus the total systematic correction is (0.4 ± 0.3) s and the final result for the neutron lifetime from our experiment is 878.5 ± 0.7_stat._ ± 0.3_syst_.

## 7. Conclusion

In the present experiment the storage time is very close to the neutron lifetime: The difference between the best-measured storage time and the neutron lifetime is about 5 s only. This gives confidence in the reliability of the result obtained.

**Fig. 4:**
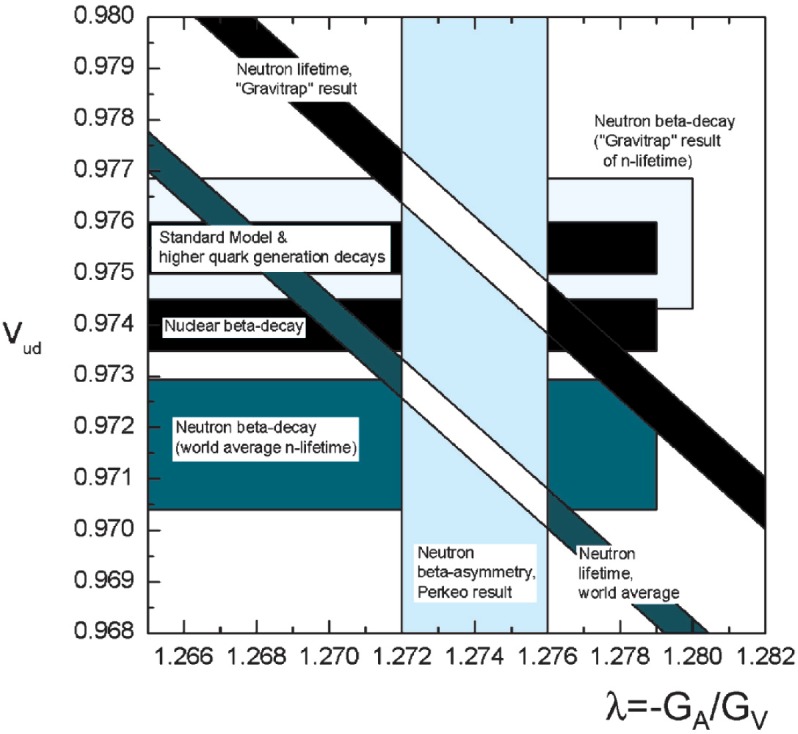
|*V*_ud_ | versus − *G*_A_ / *G*_V_. |*V*_ud_ | was derived from higher quark generation decays via $V_{\text{ud}}|=\sqrt{1-{\left|V_{\text{us}}\right|}^{2}-{\left|V_{\text{ub}}\right|}^{2}}$ predicted from unitarity, from *Ft* values of nuclear decays, and neutron *β*-decay.

The new result for the neutron lifetime can be used for the unitarity test of Cabibbo-Kobayashi-Maskawa (CKM) matrix. Figure 4 shows a plot of ***V***_ud_ versus − ***G***_A_ / ***G***_V_ from [6] with the new result for the neutron lifetime.

The new lifetime result is different from the world average value by 6.5 standard deviations, and by 5.6 standard deviations from the previous most precise result [1]. However, the new result for the neutron lifetime together with the current value for the *β* - asymmetry in neutron decay (*A*_0_ = − 0.1189(7) [2]) is in a good agreement with the Standard Model.

## Figures and Tables

**Table 1:** The systematic effects and their uncertainties

Systematic effect	Value, *s*	Uncertainty, *s*
Method of *γ* values calculation	0	0.236
Influence of *µ*(*E*) function shape	0	0.144
Spectrum uncertainties	0	0.104
Uncertainties of traps sizes (1 mm)	0	0.058
Influence of the residual gas	0.4	0.024
Uncertainty of LTF critical energy (20 neV)	0	0.004

Total systematic effect	0.4	0.3
